# Supplementary material for: Multiplexed and reproducible high content screening of live and fixed cells using Dye Drop
Source: Nat Commun. 2022 Nov 14;13:6918. doi: 10.1038/s41467-022-34536-7 (PMC9663587; doi:10.1038/s41467-022-34536-7)
Supplement: Supplementary file 1 — Supplementary Information [file 41467_2022_34536_MOESM1_ESM.pdf]

Multiplexed and reproducible high content screening of live and fixed cells using the Dye Drop method

SUPPLEMENTARY INFORMATION

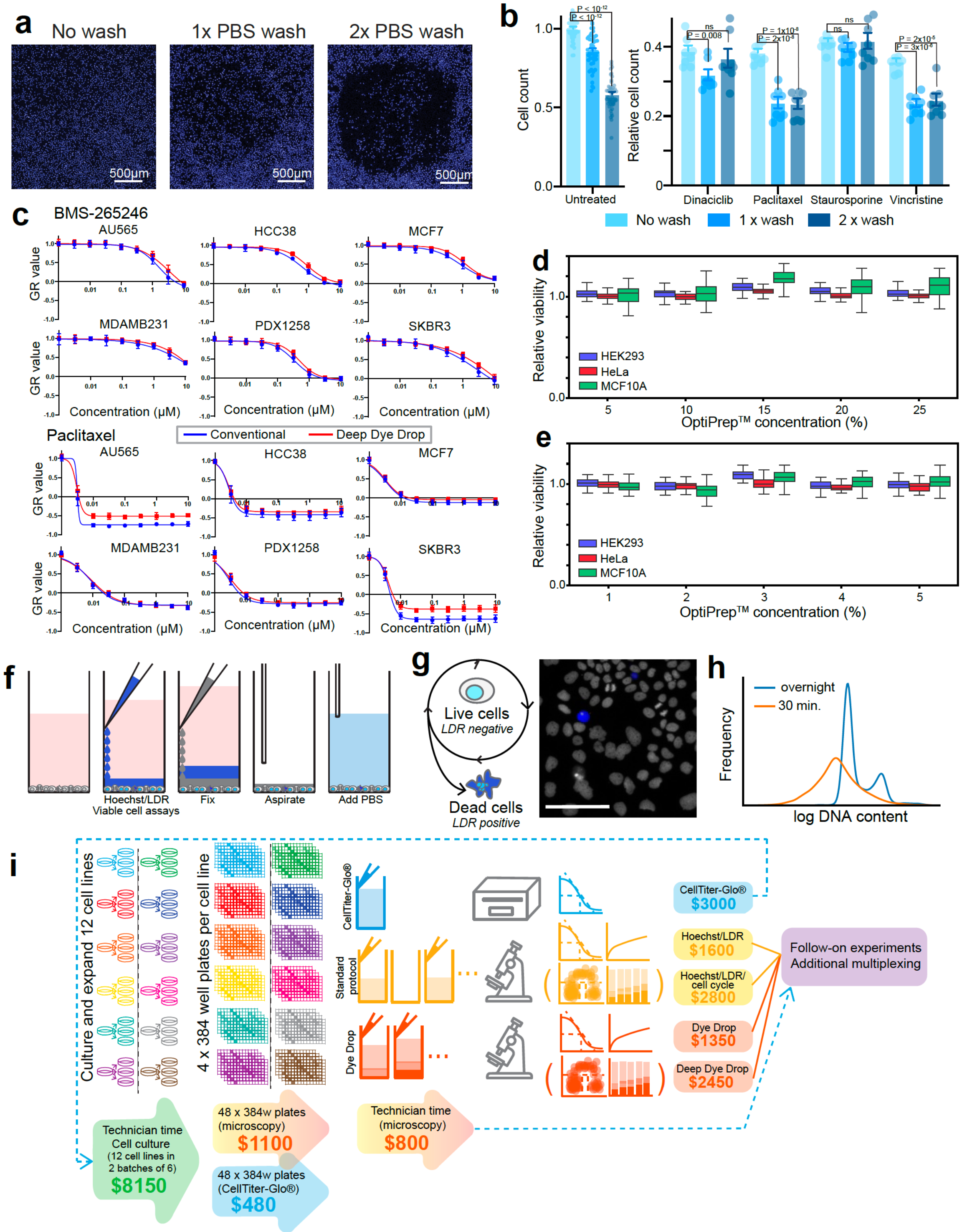

Supplementary Figure 1: Validation of assay conditions and assay schematics (a) Images of MCF 10A-H2B-mCherry cells in a well of a 384 well plate stained with Hoechst in iodixanol without prior washing and following one or two PBS washes with a robotic plate washer. The images shown are to illustrate cell loss from repeat washes, each image is representative of one well from a 384 well plate. (b) Consequences of one or two washes prior to fixation on MCF 10A-H2B-mCherry cells untreated and treated with 0.1 μM of the drugs shown for 24 h. Error bars represent the standard error of the mean of eight technical replicates from one representative biological replicate, P-values shown are from 2-way ANOVA tests corrected for multiple comparisons with Tukey's method, ns indicates not significantly different. (c) GR dose response curves for six breast cancer cell lines treated with BMS-265246 or paclitaxel for 72 h at the doses indicated and assayed by Deep Dye Drop (red lines) or by a conventional washing and staining protocol (blue lines). Error bars represent the standard deviation of the mean of biological triplicates. (d) Effects of a one-hour pulse of increasing concentrations of OptiPrep™ on the viability of HEK293, HeLa and MCF 10A cells 24 h later. The bottom and top of the box show the first and third quartiles, the bar within each box represents the median value and error bars represent the range of values of 40 technical replicates. (e) Effects of a 24 h exposure to increasing concentrations of OptiPrep™ on the viability of HEK293, HeLa and MCF 10A cells. The bottom and top of the box show the first and third quartiles, the bar within each box represents the median value and error bars represent the range of values of 40 technical replicates. (f) Dye Drop protocol steps: Hoechst and LIVE/DEAD (LDR) dye are added in 6% iodixanol (10% OptiPrep™) followed by 4% formaldehyde in 12% iodixanol (20% OptiPrep™) the contents of the well are aspirated and replaced with PBS. One well of a multi-well plate is depicted. (g) Schematic of Dye Drop staining and image showing cells stained with the Dye Drop protocol. Hoechst staining is gray-scale and LDR staining is blue. Scale bar is 100 μm. The image shown is an example of typical of Dye Drop staining as performed in 58 breast cancer cell lines in this work. (h) DNA content quantified from untreated BT20 cells stained with the Dye Drop (Hoechst 30 min) and Deep Dye Drop (Hoechst overnight (o/n)) protocols. (i) Schematic diagram of the costs associated with running a 12-cell line, 30 drug profiling experiment by CellTiter-Glo®, Dye Drop, Deep Dye Drop or equivalent assays with a conventional protocol. A portion of this panel is reproduced from our previous work[9]. Source data are provided as a Source Data file.

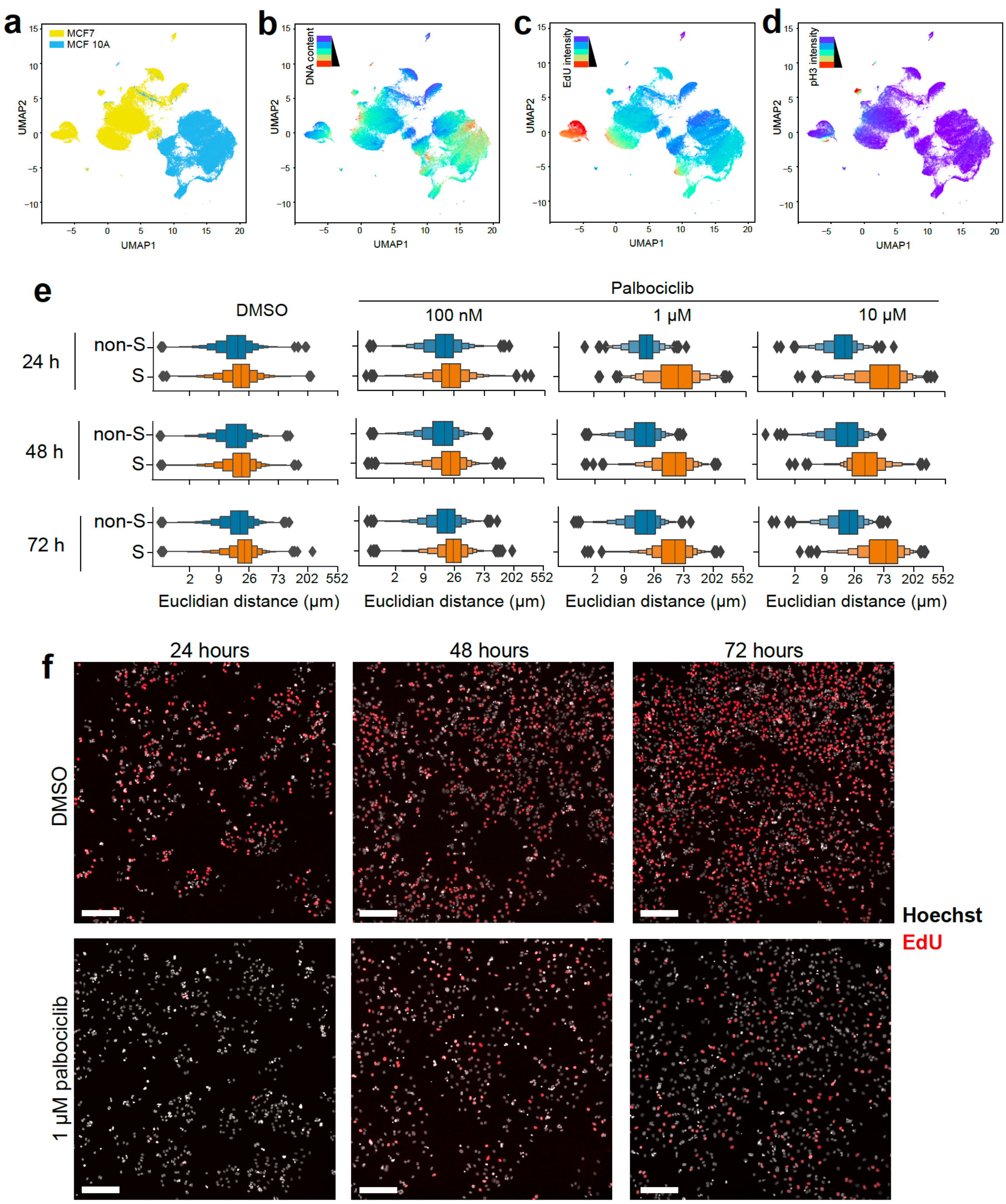

Supplementary Figure 2: Extension of Deep Dye Drop assays (a) UMAP representation of MCF7 and MCF 10A cells treated with BMS-265246 (1 µM, 10 µM), ribociclib (10 µM) or DMSO stained with Deep Dye Drop and cyclic immunofluorescence colored by cell line, (b) DNA content, (c) EdU intensity, and (d) phospho-histone H3 intensity. (e) Boxenplots showing the natural log Euclidian distance from S-phase cell to the nearest S-phase cell and between S-phase and the nearest cell assigned to any other cell cycle phase in a population of MCF7 cells treated with DMSO or palbociclib at 1 µM or 10 µM for 24, 48 and 72 h. The centerline in each plot is the median, each successive level outward contains half of the remaining data. (f) Representative images from biological duplicates performed in technical quadruplicate of MCF7 cells treated with DMSO or 1 µM palbociclib for 24, 48, or 72 h. Hoechst is shown in grayscale, and EdU in red. Scale bars are 200 µm. Source data are provided on Synapse.

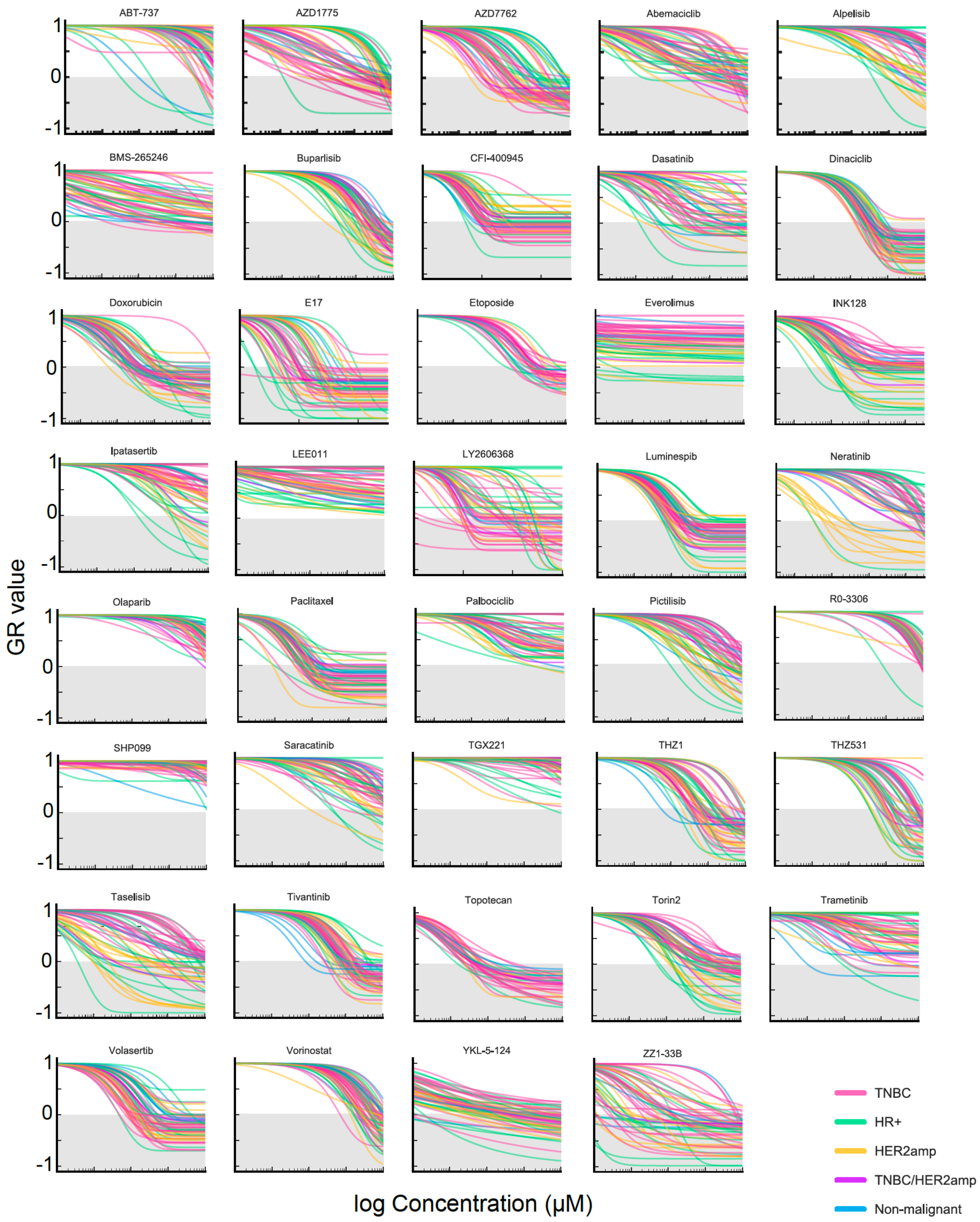

Supplementary Figure 3: GR dose response curves for select drugs in 58 cell lines GR-based dose response curves for 58 breast cancer cell lines treated with increasing concentrations of the drugs indicated for 72 hours. Cells were either stained with the Dye Drop or Deep Dye Drop assays. Each curve

represents the fit to the average of three or four technical replicates, error bars are not shown for visual simplicity. Curves are colored by clinical subtype. Source data are provided on Synapse.

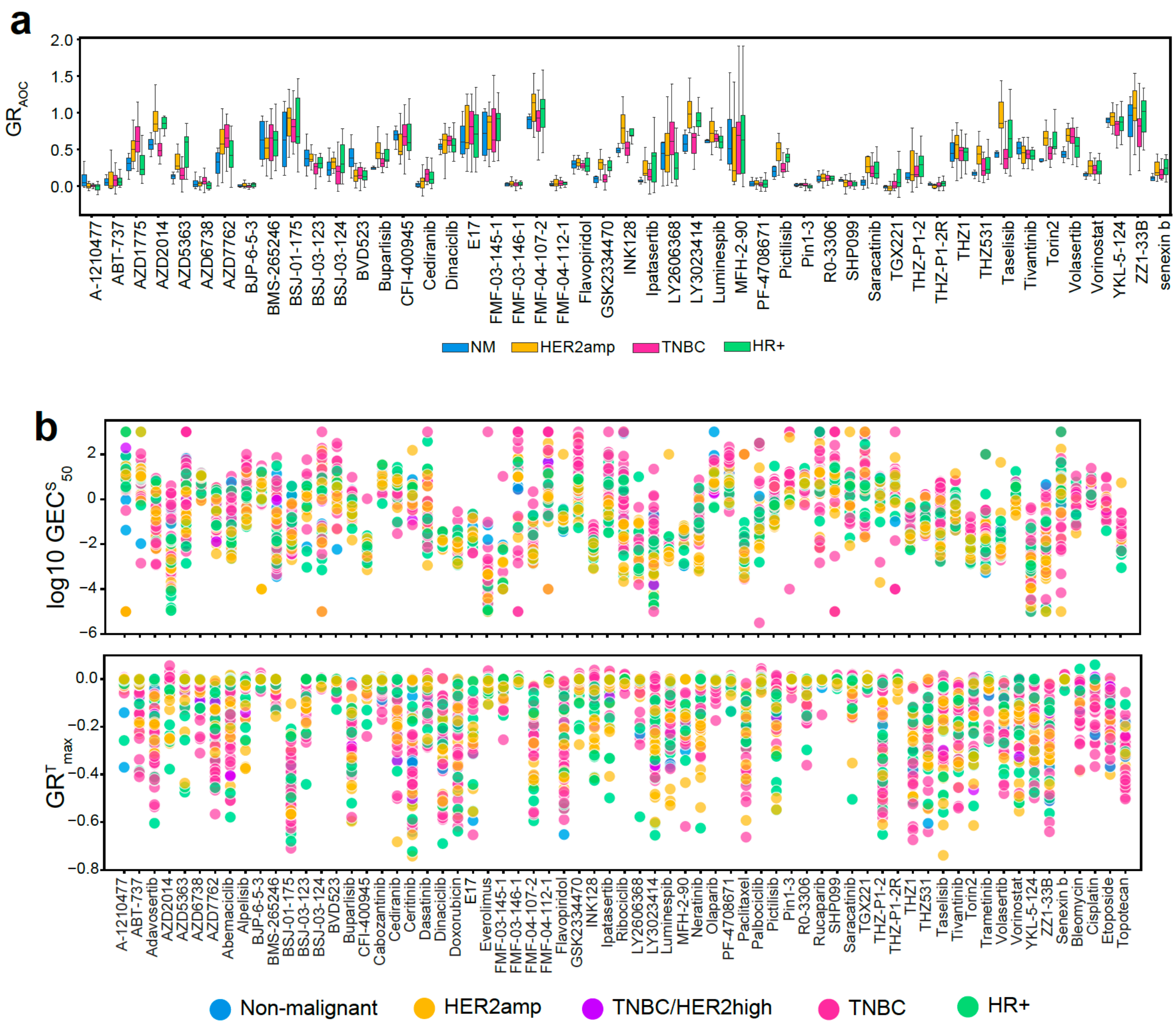

Supplementary Figure 4: GR metrics definitions and results for 67 drugs in 58 breast cancer cell lines (a) Area over the GR curves for the responses of non-FDA-approved drugs screened in 58 cell lines colored by clinical subtype (n = 5 NM, 13 $HER2^{amp}$, 13 HR^{+}, 26 TNBC). The bottom and top of the box show the first and third quartiles, the bar within each box represents the median value and error bars represent the range of values. (b) GEC^{S}_{50} and GR^{T}_{max} metrics for 67 drugs in 58 breast cancer cell lines. Source data are provided as a Source Data file.

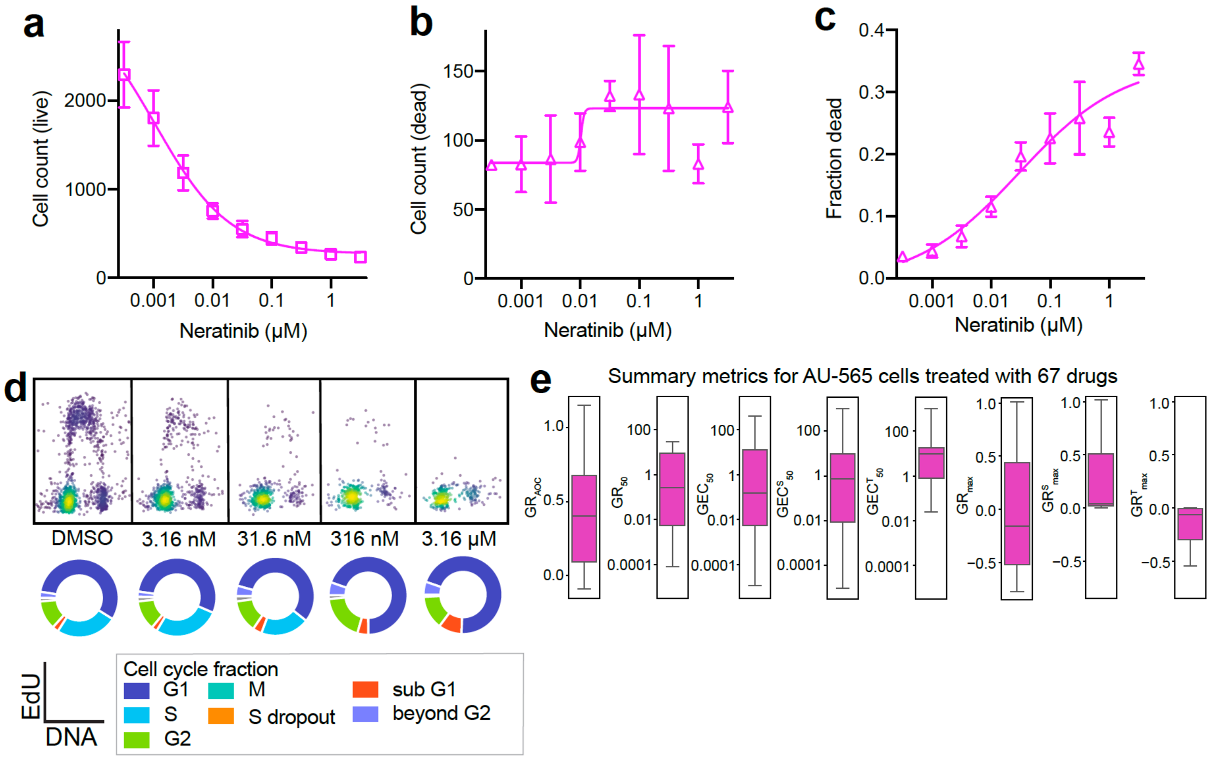

Supplementary Figure 5: Neratinib response in AU-565 cells (a) Live cell counts, (b) dead cell counts and (c) fraction of cells that are dead in AU-565 cells treated with neratinib at increasing concentrations for 72 h. Error bars represent standard deviation of the mean of technical quadruplicates. (d) Single cell EdU vs DNA content plots for AU-565 cells treated with neratinib at the concentrations indicated with corresponding circular cell cycle fraction charts. All cells from a single well of a 384 well plate are shown. (e) GR metrics across AU-565 cells treated with 67 drugs. The bottom and top of the box show the first and third quartiles, the bar within each box represents the median value and error bars represent the range of values. Source data are provided as a Source Data file.

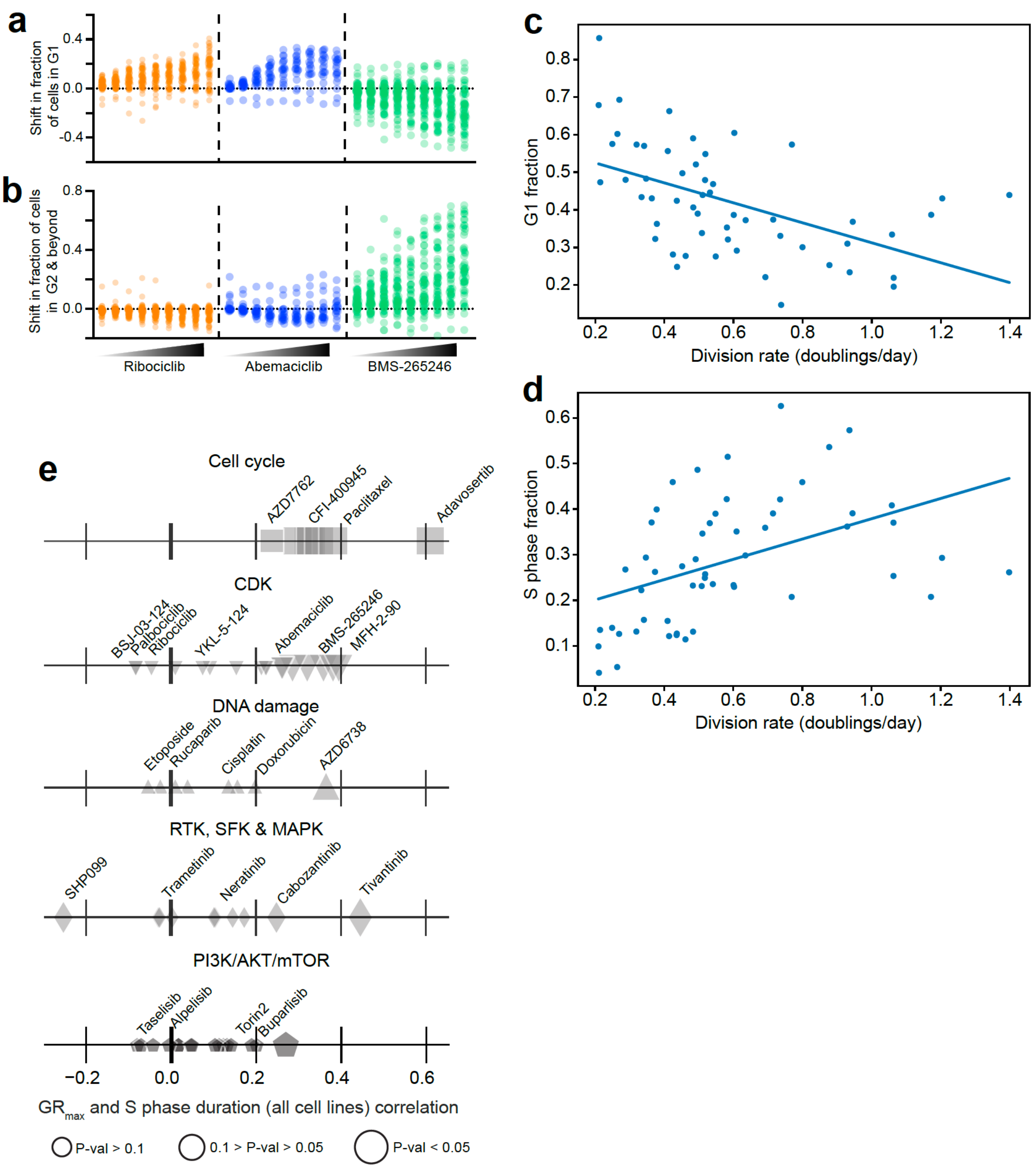

Supplementary Figure 6: Relationship between baseline cell cycle distribution and drug response (a) The effects of increasing concentrations of ribociclib, abemaciclib and BMS-265246 on the fraction of cells in G1, and (b) in G2 or with DNA content in excess of G2. (c) G1 and (d) S phase fraction with respect to division rate for 58 breast cancer cell lines. Lines of best fit are shown. (e) Spearman correlation between the duration of S-phase and the GR_{max} of the dose response curves across all cell lines for all drugs tested organized by pathway targeted. Source data are provided as a Source Data file.

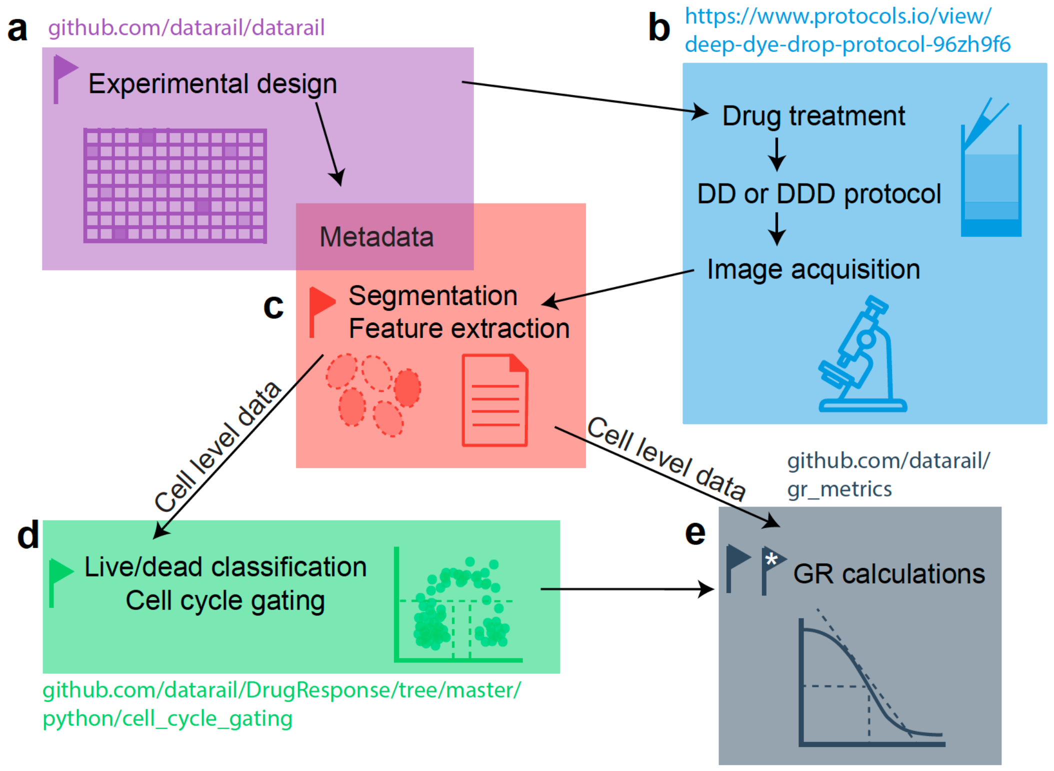

The user will be warned if a minimum number of controls have not been included.

Optimize segmentation for each cell line used.

Visually inspect gating accuracy and perform manual gating corrections as needed.

The user will be warned about large discrepancies between GR_{max} and GR_{inf} metrics.

The user will be notified of bimodal dose response curves.

Supplementary Figure 7: Overview of the components of a dose response experiment and available tools (a) Automated experimental design Jupyter notebook for the randomization of dose response studies in multi-well plates. User input: experimental parameters (drugs, concentrations, time points, cell lines etc.); output: treatment file for a D300 digital drug dispenser and the associated metadata. The user is alerted if their experimental design does not include sufficient control wells (set at 8 per cell line). (b) Following drug treatment, cells are stained and fixed using the Dye Drop (DD) or Deep Dye Drop (DDD) assay, and images are acquired on any high throughput microscope. A portion of this panel is reproduced from our previous work[9]. (c) Segmentation and feature extraction are performed and merged with the metadata. (d) Single cell level data can be gated automatically into the phases of the cell cycle. The user is presented with the gates overlaid on EdU versus DNA content scatter plots for visual inspection; should the gating be inaccurate it can be manually adjusted. Users have the option of having gates defined on negative control wells applied to treated wells, or of gating each well independently. (e) Well level data, either from feature extraction software, or summarized from single cell gating, are used to calculate GR values and fit GR metrics either using the online calculator (grcalculator.org), or a Jupyter notebook. Stacked bar graphs for cell cycle, dose response curves (GR values, GR static, GR toxic and fraction dead), and summary metrics (per drug - cell line pair for each timepoint) are output. The suite of tools is modular, each component can be used independently of the others, or jointly depending on the experiment and equipment available.

Supplementary Table 1: Metadata for antibodies used in cyclic immunofluorescence shown in Fig. 3a-b, Hoechst was included in each cycle.

Cycle	***Antigen***	***Dilution used***	***Clone***	***Fluor***	***Vendor***	***Cat #***	***Lot #***	***RRID***
1	pH3	1:1000	D2C8	488	CST	3465S	14	AB_10695860
1	pRb	1:500	D20B12	555	CST	8957S	5	AB_2728827
1	b-catenin	1:300	L54E2	647	CST	4627S	5	AB_10691326
2	PCNA	1:300	PC10	488	CST	8580S	3	AB_11178664
2	ki67	1:300	20Raj1	570	Thermo	41-5699-82	1956875	AB_11220278
2	p21	1:300	12D1	647	CST	8587S	6	AB_10892861
3	Cyclin D1	1:100	EPR2241	488	Abcam	190194	GR3282485-2	AB_2728784
3	b-actin	1:500	13E5	555	CST	8046S	3	AB_11179208
3	g-H2AX	1:300	20E3	647	CST	9720S	19	AB_10692910

REFERENCES

1. Kao, J. et al. Molecular Profiling of Breast Cancer Cell Lines Defines Relevant Tumor Models and Provides a Resource for Cancer Gene Discovery. PLoS ONE 4, e6146 (2009).
2. Neve, R. M. et al. A collection of breast cancer cell lines for the study of functionally distinct cancer subtypes. Cancer Cell 10, 515–527 (2006).
3. Chopra, S. S. et al. Torin2 Exploits Replication and Checkpoint Vulnerabilities to Cause Death of PI3K-Activated Triple-Negative Breast Cancer Cells. Cell Syst. 10, 66-81.e11 (2020).
4. Tate, J. G. et al. COSMIC: the Catalogue Of Somatic Mutations In Cancer. Nucleic Acids Res. 47, D941–D947 (2019). v96, https://cancer.sanger.ac.uk/cosmic
5. Hafner, M. et al. Multiomics Profiling Establishes the Polypharmacology of FDA-Approved CDK4/6 Inhibitors and the Potential for Differential Clinical Activity. Cell Chem. Biol. 26, 1067-1080.e8 (2019).
6. Dai, X., Cheng, H., Bai, Z. & Li, J. Breast Cancer Cell Line Classification and Its Relevance with Breast Tumor Subtyping. J. Cancer 8, 3131–3141 (2017).
7. DeRose, Y. S. et al. Tumor grafts derived from women with breast cancer authentically reflect tumor pathology, growth, metastasis and disease outcomes. Nat. Med. 17, 1514–1520 (2011).
8. Hollestelle, A., Elstrodt, F., Nagel, J. H. A., Kallemeijn, W. W. & Schutte, M. Phosphatidylinositol-3-OH kinase or RAS pathway mutations in human breast cancer cell lines. Mol. Cancer Res. MCR 5, 195–201 (2007).
9. Niepel, M. et al. A Multi-center Study on the Reproducibility of Drug-Response Assays in Mammalian Cell Lines. Cell Syst. 9, 35-48.e5 (2019).
