# Supplementary material for: Multiplexed and reproducible high content screening of live and fixed cells using Dye Drop
Source: Nat Commun. 2022 Nov 14;13:6918. doi: 10.1038/s41467-022-34536-7 (PMC9663587; doi:10.1038/s41467-022-34536-7)
Supplement: Supplementary file 2 — Editorial Assessment Report [file 41467_2022_34536_MOESM2_ESM.pdf]

nature portfolio GUIDED OPEN ACCESS

Contents of this report

- **Manuscript details**: overview of your manuscript and the editorial team.
- **Review synthesis**: summary of the reviewer reports provided by the editors.
- **Editorial recommendation**: personalized evaluation and recommendation from all 3 journals.
- **Annotated reviewer comments**: the referee reports with comments from the editors.
- **Open research evaluation**: advice for adhering to best reproducibility practices.

About the editorial process

Because you selected the **Nature Portfolio Guided Open Access option**, your manuscript was assessed for suitability in three of our titles publishing high-quality work across your field of research. More information about Guided Open Access can be found here.

Collaborative editorial assessment

Your editorial team discussed the manuscript to determine its suitability for the Nature Portfolio Guided OA pilot. Our assessment of your manuscript takes into account several factors, including whether the work meets the **technical standard** of the Nature Portfolio and whether the findings are of **immediate significance** to the readership of at least one of the participating journals in the Guided OA pilot.

Peer review

Experts were asked to evaluate the following aspects of your manuscript:

- **Novelty** in comparison to prior publications;
- **Likely audience** of researchers in terms of broad fields of study and size;
- **Potential impact** of the study on the immediate or wider research field;
- **Evidence** for the claims and whether additional experiments or analyses could feasibly strengthen the evidence;
- **Methodological detail** and whether the manuscript is reproducible as written;
- Appropriateness of the literature review.

Editorial evaluation of reviews

Your editorial team discussed the potential suitability of your manuscript for each of the participating journals. They then discussed the revisions necessary in order for the work to be published, keeping each journal's specific editorial criteria in mind.

Journals in the Nature portfolio will support authors wishing to transfer their reviews and (where reviewers agree) the reviewers' identities to journals outside of Springer Nature.

If you have any questions about review portability, please contact our editorial office at guidedoa@nature.com.

nature portfolio GUIDED OPEN ACCESS

Manuscript details

Tracking number	Submission date	Decision date
GUIDEDOA-21-00231	27 August 2021	Click or tap to enter a date.

**Title**	Multiplexed and reproducible high content screening of live and fixed cells using the Dye Drop method	**Corresponding author**	Peter Sorger<br>**Affiliation:** Harvard Medical School
**Preprint information**	There is a preprint of this manuscript posted at **bioRxiv**.	**Peer review type**	Single-blind

Editorial assessment team

**Primary editor**	**Anam Akhtar**<br>**Home Journal:** *Communications Biology*, ORCID: 0000-0002-8820-8468<br>**Email:** anam.akhtar@nature.com
**Editorial team members**	**Rita Strack**, *Nature Methods*, ORCID: 0000-0003-1845-7116<br>**Kyle Legate**, *Nature Communications*, ORCID: 0000-0003-3243-579X
**About your primary editor**	Anam received her PhD in Biomedical Sciences from Middlesex University, designing targeted liposomal constructs for selective drug delivery to HPV infected cancers. She also has research experience in genetics and the design of polymer and inorganic nanoparticles and their applications. Anam joined the editorial team of *Communications Biology* in July 2019 and is based in London office.

Editorial assessment and review synthesis

**Editor's summary and assessment**	Authors here describe the development of a method for alleviating a source of technical variability when measuring the time-dependent effects of chemical or other perturbations on cells in multiwell plates, by using iodixanol (Optiprep), an inert liquid, commonly used in radiology. Adding this liquid, such that the sequence of solutions could be made slightly denser than the last, effectively eliminated mix and wash steps.<br><br>They demonstrate the utility of this method on a panel of 58 breast cancer cell lines treated with a collection of 67 anticancer chemical compounds and use the results of the Dye Drop approach to make comparisons across breast cancer subtypes and drug classes. They demonstrate its utility by measuring DNA content and incorporation of EdU to identify cells in different phases of the cell cycle, using antibodies to quantify biomarkers of specific cell phenotypes, and applying the approach to samples obtained as a time series.<br><br>They further find that breast cancer cell lines display marked diversity of cell cycle distributions and that they vary up to 10-fold in proliferation rate, with cells of the HER2-overexpressing subtype exhibiting the slowest growth and nonmalignant cells the fastest. They also develop computational routines which present the users a series of "flags" that alert when experimental design criteria and results are suboptimal.<br><br>We found this a really nice, low tech and straightforward study which can have widespread impact on high throughput assays, which are such fundamental techniques used in labs. Using OptiPrep for high-throughput assays is new and clever. The validation is quite thorough and although new biological insight has not been obtained per se, we sent it out for review because of the neatness of the approach.
**Editorial synthesis of reviews**	All the three reviewers really liked the study and commented on the ease of the approach and the impact it can have on high-throughput assays. They gave quite an overlapping feedback. Both Reviewer 1 and Reviewer 2 asked for benchmarking and discussion on cost benefit. Reviewer 1 and Reviewer 3 commented that the impact of the manuscript would be increased if the authors demonstrated a new insight/ observation. Reviewer 1 wants development of the computational tool for easy adaptation and Reviewer 3 wants code to be updated to include new GR metric.<br><br>For further consideration at *Nature Communications*, we would ask to see further benchmarking, development of the code and to add some biological insight.<br><br>For consideration at *Communications Biology*, benchmarking and demonstration that it is more cost effective than existing techniques is important. However, the demonstration of new insight or further development of the code is not required for us.

Editorial recommendation

nature methods	Revision not invited	For *Nature Methods*, we are not inviting a revision. The focus on drug screening takes the work out of our editorial scope, which is focused on methods for basic research.
nature communications	Major revisions with extension of the study	For *Nature Communications*, we ask for the requests of Communications Biology, plus the development of code and some additional biological insight. We will not be overruling any of the reviewer concerns. This would require extensive revisions.
communications biology	Major revisions	For *Communications Biology*, although we are happy to forgo the request to demonstrate any biological insight or further development of the code than is already present in the manuscript, we do require authors to benchmark against existing techniques to show the advantage of the method. This will require additional experiments, though not very extensive, hence the decision of a major revision.

Next steps

Recommendation Summary

- Option 1: Revise for consideration at *Communications Biology*.
- Option 2: Revise for consideration at *Nature Communications*.

See the previous page for details. Nature Methods can no longer consider the manuscript because scope of the work is not relevant enough to basic research.

Revision

To follow our recommendation, please upload the revised manuscript, along with your point-by-point response to the reviewers' reports and editorial advice **using the link provided in the decision letter**. Should you need assistance with our manuscript tracking system, please contact Adam Lipkin, our Nature Portfolio Guided OA support specialist, at guidedOA@nature.com.

Revision checklist

- Cover letter, stating to which journal you are submitting
- Revised manuscript
- Point-by-point response to reviews
- Updated **Reporting Summary** and **Editorial Policy Checklist**
- Supplementary materials (if applicable)

Submission elsewhere

To a journal outside of Nature Portfolio

If you choose to submit your revised manuscript to a journal at another publisher, we can share the reviews with another journal outside of the Nature Portfolio if requested. You will need to request that the receiving journal office contacts us at guidedOA@nature.com. We have included editorial guidance below in the reviewer reports and open research evaluation to aid in revising the manuscript for publication elsewhere.

Annotated reviewer reports

The editors have included some additional comments on specific points raised by the reviewers below, to clarify requirements for publication in the recommended journal(s). However, please note that all points should be addressed in a revision, even if an editor has not specifically commented on them.

**Reviewer #1**	
**Reviewer #1**	This reviewer has not chosen to waive anonymity. The reviewer's identity can only be shared with representatives of an established journal editorial office.
**Reviewer #1 expertise**	microscopy-based high content screening
**Editor's comments about this review**	The reviewer really appreciated the method, which he found straightforward, fascinating and useful to the field. However, he raised some very pertinent points on the need to demonstrate versatility, cost effectiveness and insight, which can elevate the study even more.
**Reviewer #1 comments**	
**Overview**	In their manuscript, Mills et al. describe a new method for processing cells after treatment in microtiter multi-well plates. In essence, the central claim is that the application of Optiprep, a density gradient medium, can optimize robot-assisted liquid handling in high throughput screens two-fold. First, through density governed displacement of the less dense medium, highly agitating pipetting steps are avoided to preserve the in situ adherence, morphology, and cell setup within a well. Second, since density displacement does not require large volumes of, for example, staining solutions, expensive reagents are saved, and costs can become significantly reduced.<br><br>I find this approach to the optimization of high throughput screening campaigns intriguing and original. Furthermore, I find the gentleness of the approach specifically exciting and relevant to a large field of assays used in drug discovery, functional genomics, genome editing, and many more. Yet, I am convinced that discussion and benchmarking of the methodological aspects of the author's ideas beyond analysis known from other studies that introduced morphological profiling leave room for improvement of the manuscript.

Specific comments		
**#**	**Reviewer comment**	**Editorial comment**
1	**Impact:** Other image-based high throughput profiling campaigns also found that changes in cell cycle distributions, among other derivative features, can be much more informative than, for example, ATP levels of cells (reviewed in Boutros et al. 2015, Caicedo 2016, original in Gut, Nat. Meth., 2015 among others). Here, the authors miss a real opportunity to convince that their method could change the way high content screens are performed going forward. For example, I could imagine that certain primary cultures, co-culture assays, and more complex procedures can benefit immensely from this methodological advance. Thus, I think the manuscript could significantly gain impact if the advance of information content and assay versatility are demonstrated more extensively.	**While this is indeed a very useful comment and can certainly elevate the method's utility, showing assay versatility is not mandatory for consideration at *Communications Biology*.**<br>**For *Nature Communications* we ask that you address this point to increase the assay versatility.**
2	While the scale of the screen at which the method was demonstrated represents an impressive effort, I was surprised that the detailed discussion of the DyeDrop methodology was carried out in MCF10a and MCF7 cell lines. Both are cell lines, more or less accessible by traditional liquid handling methods.	**Please clarify the choice of the cell lines employed in this study.**
3	I am further not sure if the findings the authors discuss from their drug screening campaign in a panel of 50 cell lines could not be recovered by conventional means of liquid handling. Accurate side-by-side comparisons of the same experiments carried out using traditional methods and Dye drop under fair and comparative conditions would greatly add to the manuscript's merit.	**Both journals would require benchmarking as requested by this reviewer.**
4	In addition, while I find the compatibility with CyCIF attractive, I could not see how this could not have been done without DyeDrop. Even though I find this highly multiplexed image-based Cytometry exciting and want to see its application in solving more drug mechanisms, I failed to see its relevance for this particular manuscript. Maybe, the manuscript could be improved if the authors could explain and demonstrate how mechanistic insights	**While this doesn't need to be extensively addressed for *Nature Communications*, we do ask for further biological insight which could be addressed here.**<br>**Just a textual discussion for this point will suffice for**

	can be drawn from the experiment shown.	***Communications Biology***.
**5**	Also, I'd recommend discussing how DyeDrop improved assay performance and/or cost in the discussion section of the manuscript.	**Please do discuss how dye Drop method is more effective than existing methods in terms of cost and performance. This is a requirement for either journal.**
**6**	Readers might be genuinely curious if the authors also found new observations in their large panel of cell lines, e.g., some unexpected interactions between the drugs and the cell lines.	**The editors at *Nature Communications* request that you please address this comment in full.**<br>**This point is optional to address for *Communications Biology*.**
**7**	On page 15, I was surprised to find a discussion of GO:term enrichment from GSEA. Where did the data for this come from? How is this related to the screen or the Dye Drop method?	**Editors of both journals ask that you please explain this point in the manuscript.**
**8**	Why is the computational pipeline for single-cell Phenomics just a side note in the manuscript, while the field of high-content cell profiling still lacks various methods in this area. A greater discussion of the approaches used, the findings, and the complications would undoubtedly be relevant for the field.	**Editors of both journals ask that you please discuss this point in the manuscript.**
**9**	The description of the segmentation and data pre-processing pipelines also seemed superficial, and the manuscript would benefit from a more detailed description.	**Editors of both journals ask that you please discuss this point in the manuscript.**
**10**	Why did the authors switch from a 2-way ANOVA to 2-tailed t-test? Technically, these are similar, if not the same, but the variance in descriptions is confusing.	**Editors of both journals ask that you please use the same statistical test for consistency.**
**11**	In Figure 5, it is hard to extract differences in Subtype specific drug responses by eye. Summary stats would help to disentangle more subtle interactions of drugs and cell backgrounds.	**Editors of both journals ask that you please address this point in the manuscript. Specifically, please make the summary statistics available via an online repository or supplementary information and provide an analysis of these interactions to the level possible.**

**12**	While I welcome that the authors provide all code and data to reproduce the method and analyses, I am convinced that the tool suite needs to be developed further from a BoS set up to a more elaborate toolbox for more accessible and widespread adoption.	**For *Nature Communications* we would like you to address this point to allow for more widespread adoption of your method.**<br>**While this will make your method more accessible to wider audience, further developing your tool suite is not mandatory for consideration at *Communications Biology*.**

Reviewer #2

**Reviewer #2**	This reviewer has not chosen to waive anonymity. The reviewer's identity can only be shared with representatives of an established journal editorial office.
**Reviewer #2 expertise**	biochemical and cellular pharmacology
**Editor's comments about this review**	Reviewer 2 has provided an overall positive assessment of the paper, but also raises the point on benchmarking and cost effectiveness discussion as raised by the previous reviewer. Please address all the points of this reviewer.

Reviewer #2 comments

**Overview**	**Significance:**<br>The manuscript by Mills et al essentially presents two significant sets of results. First, the authors present a novel method for efficiently staining cells in high-density screening plates that enables multiplexed staining while minimizing the disruption of the cell monolayer or, importantly, loss of specific weakly-adhered sub-populations of cells. These are important factors in ensuring consistent and reliable data generation for profiling small-molecule mechanisms of action, for example in chemogenomic profiling of cell line panels. The context of the problem that is addressed is well described.<br>In the second part of the manuscript, the Dye Drop method is used to demonstrate the application of multiplexed high-content imaging in the study of differential cellular responses to cell cycle kinase-targeted inhibitors. This work builds on the extensive prior work by this group in developing descriptive metrics for differential mechanisms of response as well as potency for drug treatment.<br>As a spin-off of these proof-of-concept studies, a significant body of data describing

	detailed effects on cell cycle parameters of a large number of approved and investigational drugs across a large panel of breast cancer cell lines is provided as a resource for the community.<br>**Impact:**<br>The method described is likely to be quite impactful in the field of high-content screening. This is an improvement on one of the very fundamental and ubiquitous processes.<br>The drug-response data presented will also be impactful both as a resource providing insights into specific mechanisms of action for widely-used drugs, and as a demonstration of the value of higher-dimensional profiling in chemogenomic studies.<br>The authors continue their valuable mission of advocating for more reliable, sophisticated and meaningful data analysis.

Specific comments

#	Reviewer comment	Editorial comment
1	All the data are well-presented and robust. However the potential impact of the Dye Drop method, in terms of uptake by other investigators, could be enhanced by further comparison with existing methods.	**As mentioned above, benchmarking is essential in the revised version for either journal.**
2	Figure 1b is actually the only piece of data showing superiority of the Dye Drop method, in that a no-wash method avoids loss of cells. That in itself is not a novel observation. The claim that eliminating wash steps avoids selective loss of mitotic and/or dying/dead cells is intuitively obvious, but there is no head-to-head demonstration that the data is better or more reproducible.	**Kindly address this point and show the superiority of the method by appropriate comparisons, as also requested by reviewer #1.**
3	There are also mentions and claims that the method reduces reagent costs, and it would also presumably significantly improve processing efficiency and throughput. Some attempt to quantify these saving in terms of cost, materials and time relative to the standard wash/add/wash/add method would be very beneficial. This is speaking from the point of view of a practitioner making the case for implementing the new method.	**Editors of both journals ask that you please address this point in the manuscript.**

Reviewer #3	
Reviewer #3	This reviewer has not chosen to waive anonymity. The reviewer's identity can only be shared with representatives of an established journal editorial office.
Reviewer #3 expertise	microscopy, cell-based assays
Editor's comments about this review	Reviewer 3 also likes the method and comments on the potential impact. They appreciate that the code and data is made freely available and have some important points on the computational and theoretical part of your study.
**Reviewer #3 comments**	
Overview	**Summary:**<br>The manuscript by Mills et al. entitled, "Multiplexed and reproducible high content screening of live and fixed cells using the Dye Drop method" describes the development of a method for alleviating a source of technical variability when measuring the time-dependent effects of chemical or other perturbations on cells in multiwell plates, commonly used in high throughput experiments. This work demonstrates the importance of obtaining time-dependent measurements of drug effects on cells at the individual cell level and highlights their novel approach to reduce the technical variability associated with this endeavor.<br>Their Dye Drop method cleverly leverages an inert liquid (iodixanol) that is compatible with maintaining cell viability and can be prepared at different densities to allow sequential administration of different aqueous reagents to label cellular constituents and processes that can be subsequently quantified at the single-cell level by fluorescence microscopy. This method aims to reduce the volumes of reagents necessary for each step, decrease the amount of cell loss that occurs with repeated fluid transfers, and enhance accuracy and reproducibility of the experimental results. The authors use a panel of 58 breast cancer cell lines treated with a collection of 67 anticancer chemical compounds and use the results of the Dye Drop approach to make comparisons across breast cancer subtypes and drug classes. The authors use several different approaches to demonstrate the utility of the Dye Drop (and Deep Dye Drop) method, including measuring DNA content and incorporation of EdU to identify cells in different phases of the cell cycle, using antibodies to quantify biomarkers of specific cell phenotypes, and applying the approach to samples obtained as a time series.<br>The authors further attempt to distinguish the time-dependent effects of drugs on cytostasis and cytotoxicity by extracting two novel metrics related to their original formulation of GR (GR_static and GR_toxic). They describe the mathematical

	derivation of these metrics and how they relate to the overall GR and proceed to obtain values for these (and other) metrics from the large breast cancer panel treated with the compound library. From these data they depict a variety of observations that were enabled by their approach, including the apparent differences in efficacy of specific kinase inhibitors on molecular subtypes of breast cancer cell lines. They made all of the resultant extracted metrics publicly available (on Synapse and https://labsyspharm.shinyapps.io/HMSLINCS_BRCA_Browser/), and refer to their (previously deployed) publicly available computational code (DataRail and GRmetrics) for performing various steps in the process.<br>**Impact:**<br>This paper will add substantially to the growing body of evidence in support of using time-resolved, single-cell measurements to better understand cellular responses to chemical perturbations, especially anticancer drugs. It describes a novel set of drug response data and well curated computational tools, all of which are publicly available for use by the broader scientific community. The authors make many observations from their data, including that inhibitors with different target specificity toward CDKs affect a panel of breast cancer cell lines in different ways, with some inhibitors having significant variance in potency across the panel but minimal cytotoxicity whereas others were nearly equipotent across all cell lines but varied dramatically in how much cell death was elicited. *Nature Communications* or *Communications Biology* would be an appropriate journal to publish this work.<br>The new work being presented in this manuscript can be separated into three categories: 1) the Dye Drop approach that enhances the generation of single-cell data of drug responses using multiplexed fluorescence microscopy, 2) the generation of a large, publicly available dataset that includes a visualization tool, and 3) the theoretical basis and computational code used to interpret the data.

Specific comments

#	Reviewer comment	Editorial comment
1	The authors do a reasonable job of providing examples of the utility of the Dye Drop approach. However, a distinct advantage of the use of microscopy to quantify molecular species at the single-cell level is the maintenance of the spatial relationship among cells in a population. The data generated using the authors' approach should contain a plethora of spatial information, but the authors do not attempt to leverage it in any way—or even describe its potential utility. Based on the data presented, it is unclear how the Dye Drop method provides information beyond what could be obtained using high throughput mass or	**Again, kindly benchmark your method and address the related points mentioned here, as requested by the other reviewers as well.**

	flow cytometry-based techniques. There should be some attempt to compare and contrast these approaches, preferably with a direct experimental comparison. For example, using the Dye Drop method, the quantification of DNA content from fluorescence images is log transformed. This is inconsistent with the quantification of DNA content in linear scale by flow cytometry. The authors should provide some rationale for their transformation and how it may impact the interpretation. A direct comparison of their results to those obtained by flow cytometry would be helpful.	
**2**	The authors provide a substantial amount of new data using the Dye Drop approach in the manuscript and make them publicly available as a web-accessible resource to the scientific community. This is a major strength of the work.	
**3**	The standardized, detailed approach (point 1) and open-source code are models of open science toward which the broader scientific community is striving. Quantitative methods, at their core, should provide the framework for others to generate similar data under different conditions (e.g., on different cell lines and drugs.) However, the interpretation of the data presented in the current study, including the theoretical basis and computational tools provided to address these new data, are incompletely or confusingly described. This also leads to the challenge of interpreting the results in biological terms. The following specific deficiencies should be addressed:<br>a) The mathematical formulas of the new GR-derived metrics (GR_static (GR^S) and GR_toxic (GR^T)) use inconsistent notation that makes it harder to understand. For example, the derivation of k_s and k_d are determined over a range of time and thus should not be a function of time, i.e., k_s(c) rather than k_s(c,t). But the total live cell number should be dependent on drug concentration, so it is unclear why x(t) rather than x(c,t) is on the right side of the equations on line 517. On line 536, it seems “where k_d(c) << 0” should instead be, “where k_d(c) >> 0,” since the death rate of cells cannot be less than 0 (i.e., k_d(c) ≥ 0).	**All of the points mentioned in this comment are important to fully address for further consideration in either journal.**

b) On lines 305–6 the authors write, “With data comprising only two time points, the model necessarily assumes that ks and kd are constant over the course of the experiment.” However, the model is *formulated* such that ks and kd are constant, independent of whether more measurements are obtained or not.

c) The authors refer to “decomposing” the GR value into the GR^S and GR^T metrics (line 309) and state that the “overall GR value is not mathematically equal to the sum of GR^S and GR^T but approximately to their product” (lines 303–4). This relationship (product rather than sum) is nonintuitive. Is this being done simply for visualization or is there some underlying rationale for scaling these values this way? How well can GR curves from various cell line/drug conditions be predicted from data using the equation (GR^S+1)*(GR^T+1)?

d) The authors seem to use the terms “proliferation”, “viability” and “S-phase” interchangeably. For example, on line 154 the authors state that, “...OptiPrepTM did not affect cell proliferation...” and refer to Supp Fig 1b and 1c which have “Relative viability” on the y-axis. Similarly, on lines 232–234 the authors state, “Proliferation was inhibited for 24 hours after drug exposure...” and refer to Fig. 3c-e; however, the panels in Fig 3c–e each have “Count of S-phase cells” as the y-axis label. The use of these various terms should be used more precisely and appropriately with respect to the quantitative results. For example, while the proportion of cells in S-phase is indicative of cells that have past the G1/S transition and have generally committed to completing the cell division cycle ending in mitosis, the proportion itself cannot be used to accurately determine a proliferation rate (i.e., a change in cell number over time) or even whether a cell will actually divide in the future; likewise, the term viability is generally used as a static metric, i.e., a single point in time. The authors should ensure the appropriate terms are used when referring to the presented data.

Minor issues with figures: please address all points		
**4**	Fig 3b, UMAP representation of multiparametric Dye Drop data. No description of all the measured variables (features) that were used to generate the UMAP was provided.	
**5**	Fig 4a: The title of one of the panels is, "AU-565 cells exposed to 67." It appears that "drugs" should be added to end.	
**6**	Fig 4c. How were molecular features/classifications determined for each cell line? How did the data specifically generated in your studies correspond to these classifications? For example, GR_AOC is shown, but what were pRb levels in respective proficient or deficient groups? Was any pRb detected in cell lines considered deficient? It is also unclear why some of the drug names are in bold.	
**7**	Fig 5b: For at least one plot for each axis, values of the tick marks should be indicated. Of particular importance is where the value of 0 is on the y-axis.	
**8**	Fig 6: What are the units of Growth rate in Fig 6a? day^-1 (base e)? doublings/day? The authors should find a way to better distinguish between what they refer to as "Growth rate," which appears to be an actual rate (per unit time) and GR, which appears to be dimensionless.	
**Reproducibility**		
**9**	While all the data (cell measurements, calculated GR values and extracted metrics) are publicly available at Synapse.org and the GRmetrics code is freely available on GitHub.com, the code (originally designed to calculate GR) has not been updated to obtain the new metrics defined in this study (e.g., GR^S and GR^T).	**Further update of the code is not needed for consideration at *Communications Biology*.**<br><br>**For *Nature Communications* we would ask you to further update the code to obtain the new metrics.**
**10**	To facilitate a broader use of these resources and facilitate reproducibility, the authors should prepare a small demonstration (for example, a Jupyter notebook or R Markdown file) of how to use the GRmetrics function to	**Editors of both journals ask that you kindly address this point by providing a user guide as**

	process a subset of the data presented in this work (like Neratinib treated AU-565 cells) and graphically depict the result (like Fig 4f). Ideally, this would include an example of the inputs and outputs of the various functions being called starting from raw data (or refer to prior work that describes each relevant step) for each type of analysis (CycIF, cell cycle assessment, GR, etc.). As it is currently presented, it is very challenging to understand what measurements are being used to generate the various metric values being presented in the figures (even with all of the values being provided on Synapse.)	**requested by the reviewer.**

Open research evaluation

Data availability
**Data availability statement**
Thank you for including a Data Availability statement. Kindly separate the Code Availability as a separate section.
**Other data requests**
All source data underlying the graphs and charts presented in the main figures must be made available as Supplementary Data (in Excel or text format) or via a generalist repository (eg, Figshare or Dryad). This is mandatory for publication in a Nature Portfolio journal, but is also best practice for publication in any venue.<br><br>The following figures require associated source data: Fig 1a,b; 2a; 3c,d,e; 4b,c,f and 6a.
Please ensure that datasets deposited in public repositories are now publicly accessible, and that accession codes or DOI are provided in the "Data Availability" section. As long as these datasets are not public, we cannot proceed with the acceptance of your paper. For data that have been obtained from publicly available sources, please provide a URL and the specific data product name in the data availability statement. Data with a DOI should be further cited in the methods reference section
**Reporting & reproducibility**
Nature Portfolio journals allow unlimited space for Methods. The Methods must contain sufficient detail such that the work could be repeated. It is preferable that all key methods be included in the main manuscript, rather than in the Supplementary Information. Please avoid use of “as described previously” or similar, and instead detail the specific methods used with appropriate attribution.
We encourage you to share your step-by-step experimental protocols on a protocol sharing platform of their choice. The Nature Portfolio’s Protocol Exchange is a free-to-use and open resource for protocols; protocols deposited in Protocol Exchange are citable and can be linked from the published article. More details can be found at www.nature.com/protocolexchange/about

Please state in the legends how many times each experiment was repeated independently with similar results. This is needed for all experiments, but is particularly important wherever results from representative experiments (such as micrographs) are shown. If space in the legends is limiting, this information can be included in a section titled "Statistics and Reproducibility" in the methods section. Legends requiring revision: Please note that this information is missing in the legends of figures 1d, 2e, supplementary figure 1e.
Legends requiring revision: 1. Please note that the box plots need to be defined in terms of minima, maxima, bounds of box and whiskers and percentile in the legends of figures 4c, 6a (bottom panel), supplementary figures 3a, 4d.
Legends requiring revision: 1. Please indicate the statistical test used for data analysis and where appropriate, please specify whether it was one-sided or two-sided and whether adjustments were made for multiple comparisons, in the legend of supplementary table 1. 2. Please note that the exact p value should be provided, when possible, in the legends of figures 1b, 4c. 3. Please indicate what '*'/ '**' represent; if these represent p values, please indicate the statistical test used and where appropriate, specify whether it was one-sided or two-sided and whether adjustments were made for multiple comparisons and the exact p value in the legend of figure 6a (bottom panel).
Panels requiring revision: 1. Please note that the scale bar is missing for figure 3a (right panel). 2. Please note that the scale bar needs to be defined for figures 1d (right panel), 2e (right panel), supplementary figure 1e.
Please ensure that data presented in a plot, chart or other visual representation format shows data distribution clearly (e.g. dot plots, box-and-whisker plots). When using bar charts, please overlay the corresponding data points (as dot plots) whenever possible and always for n ≤ 10. (Please see the following editorial for the rationale behind this request and an example https://www.nature.com/articles/s41551-017-0079). Panels requiring revision: Please note that data presentation has to be revised to comply with our policy in figures 1b, 3c-e (top-left panels), supplementary figures 1b-c.
Wherever statistics have been derived (e.g. error bars, box plots, statistical significance) the legend needs to provide and define the n number (i.e. the sample size used to derive statistics) as a precise value (not a range), using the wording "n=X biologically independent samples/animals/cells/independent experiments/n= X cells examined over Y independent experiments" etc. as applicable. Legends requiring revision: 1. Please note that this information is missing in the legends of figures 4c, 6a (bottom panel), supplementary figures 3a, 4d.
